# Differential effects of cisplatin in proximal and distal renal tubule epithelial cell lines

**DOI:** 10.1038/sj.bjc.6990047

**Published:** 1999-01

**Authors:** R Kröning, D Katz, A K Lichtenstein, G T Nagami

**Affiliations:** Hematology/Oncology Section, Medical and Research Services, West Los Angeles VA Medical Center and UCLA School of Medicine, Los Angeles, CA, USA; Nephrology Sections, Medical and Research Services, West Los Angeles VA Medical Center and UCLA School of Medicine, Los Angeles, CA, USA

**Keywords:** renal tubule epithelial cell lines, cisplatin, carboplatin, nephrotoxicity, transport, apoptosis

## Abstract

Pathological studies suggest that cisplatin injures different portions of the nephron to different extents. To investigate this issue further, we examined the cytotoxicity and uptake of cisplatin in cell lines derived from S_1_ and S_3_ proximal tubule and distal convoluted tubule segments isolated from a mouse carrying the SV40 large T-antigen transgene. S_1_ cells displayed the highest sensitivity to cisplatin cytotoxicity, followed by S_3_ and distal convuluted tubule (DCT) cells. These differences in cytotoxicity did not correlate with differences in cisplatin uptake. Cytotoxic concentrations of cisplatin triggered apoptosis in all three cell lines. Although BAX and BCL-2 expression was similar among the three cell lines, the expression of the anti-apoptotic protein, BCL-X_L_, was significantly lower in S_1_ cells than in S_3_ and DCT cells, and this may have contributed to the heightened sensitivity of S_1_ cells. Cisplatin transport characteristics demonstrated a saturable component of cisplatin uptake and differences in apparent *K*_M_ and *V*_max_ values among the three cell lines. The three cell lines were 43- to 176-fold more sensitive to cisplatin than to carboplatin. This distinction between the two drugs could not be fully explained by differences in the uptake rates of carboplatin and cisplatin. We conclude that cells from different portions of the nephron display different sensitivities to cisplatin, different transport characteristics for cisplatin and different levels of expression of BCL-X_L_. In addition, the relative resistance of renal cells to carboplatin vs cisplatin is mostly due to the differential effects that follow internalization. © 1999 Cancer Research Campaign


*British Journal of Cancer* (1999) **79**(2), 293–299
© 1999 Cancer Research Campaign

### Differential effects of cisplatin in proximal and distal renal tubule epithelial cell lines

**R Kröning[1], D Katz[1], AK Lichtenstein[1] and GT Nagami[2]**

[1]Medical and Research Services, Hematology/Oncology and [2]Nephrology Sections, West Los Angeles VA Medical Center and UCLA School of Medicine, Los Angeles, CA, USA

**Summary** Pathological studies suggest that cisplatin injures different portions of the nephron to different extents. To investigate this issue further, we examined the cytotoxicity and uptake of cisplatin in cell lines derived from $S_1$ and $S_3$ proximal tubule and distal convoluted tubule segments isolated from a mouse carrying the SV40 large T-antigen transgene. $S_1$ cells displayed the highest sensitivity to cisplatin cytotoxicity, followed by $S_3$ and distal convuluted tubule (DCT) cells. These differences in cytotoxicity did not correlate with differences in cisplatin uptake. Cytotoxic concentrations of cisplatin triggered apoptosis in all three cell lines. Although BAX and BCL-2 expression was similar among the three cell lines, the expression of the anti-apoptotic protein, BCL-$X_L$, was significantly lower in $S_1$ cells than in $S_3$ and DCT cells, and this may have contributed to the heightened sensitivity of $S_1$ cells. Cisplatin transport characteristics demonstrated a saturable component of cisplatin uptake and differences in apparent $K_M$ and $V_{max}$ values among the three cell lines. The three cell lines were 43- to 176-fold more sensitive to cisplatin than to carboplatin. This distinction between the two drugs could not be fully explained by differences in the uptake rates of carboplatin and cisplatin. We conclude that cells from different portions of the nephron display different sensitivities to cisplatin, different transport characteristics for cisplatin and different levels of expression of BCL-$X_L$. In addition, the relative resistance of renal cells to carboplatin vs cisplatin is mostly due to the differential effects that follow internalization.

**Keywords:** renal tubule epithelial cell lines; cisplatin; carboplatin; nephrotoxicity; transport; apoptosis

Cisplatin is one of the most effective anti-tumour agents for the treatment of a variety of human solid tumours, including testicular, ovarian, bladder, lung and head and neck cancers (Loehrer and Einhorn, 1984). The major dose-limiting side-effect of cisplatin is nephrotoxicity (Colvin, 1993). Pathological studies have suggested that different portions of the kidney display different degrees of damage in response to cisplatin in vivo (Blachley and Hill, 1981; Brady et al, 1990; Leibbrandt et al, 1995). Cisplatin is taken up by renal tubular cells, reaching its highest concentrations in the renal tubular cells of the inner cortex and outer medulla, which includes cells of the proximal tubule and thick ascending limb of Henle (Blachley and Hill, 1981; Chopra et al, 1982). However, distal convoluted tubules may also be involved in cisplatin-induced kidney injury (Gonzales-Vitale et al, 1977; Chopra et al, 1982).

Despite two decades of clinical use as a chemotherapeutic drug, the mechanisms by which cisplatin causes kidney damage and different degrees of damage within different portions of the kidney are not well understood. The development of in vitro culture of immortalized but non-transformed renal tubular epithelial cells provides a model to address these problems. Our approach was based upon studies by Brinster and colleagues suggesting the feasibility of creating immortalized cell culture lines from transgenic mice expressing certain portions of the early SV40 virus DNA (Palmiter et al, 1985). We dissected specific portions of the nephron of a Brinster large T transgenic mouse and cultured the segments separately on collagen-coated wells. This resulted in the production of a unique set of immortalized but non-transformed renal tubular epithelial cell culture lines: the cell lines $S_1$ and $S_3$, derived from early and late portions of the proximal tubule, respectively, and DCT cells, derived from the distal convoluted tubule. The cultured cells display contact inhibition as well as characteristics similar to those observed in corresponding freshly dissected nephron segments. For example, $S_1$ and $S_3$ cells are gluconeogenic, produce increased amounts of ammonia in response to acid challenge and respond appropriately to hormones. DCT cells display parathyroid hormone sensitivity (Nagami et al, 1990, 1991, 1992; Kaunitz et al, 1993).

In this study, we show that cisplatin and carboplatin affect these cell lines differentially in a fashion that is similar to in vivo data. Cisplatin and carboplatin cytotoxicity and uptake differ among the three cell lines. We show that the rate of transport of cisplatin is not a determinant of the differential sensitivity among renal cells. Also, for the first time, we present data that suggest that there is a saturable component of cisplatin uptake. In all cell types, apoptosis occurs in cisplatin-induced cell death.

*Received 29 January 1998*
*Revised 6 May 1998*
*Accepted 8 May 1998*

*Correspondence to:* GT Nagami, Nephrology Section (111L), VA Medical Center, West Los Angeles, 11301 Wilshire Blvd, Los Angeles, CA 90073, USA

### MATERIALS AND METHODS

### Cell culture

$S_1$, $S_3$ and DCT cell lines were established as described previously (Kaunitz et al, 1993). $S_1$ cells were derived from the earliest proximal tubule segment, $S_3$ cells from the late straight proximal tubule and DCT cells from the early distal convoluted tubule from a large T-antigen transgenic mouse. Cells were grown in cell culture dishes or multiwell plates in Dulbecco's modified Eagle medium (DMEM)–Ham's F12 medium (Sigma) supplemented with 7% heat-inactivated fetal bovine serum, 5 μ $ml^{-1}$ insulin and 5 ng $ml^{-1}$ sodium selenite at 37°C in 5% carbon dioxide/35% oxygen. All cell lines tested negative for mycoplasma contamination.

### Cytotoxicity assay

Confluent monolayers were treated with various concentrations of cisplatin (Sigma) and carboplatin (Bristol-Myers Squibb, Princeton, NJ, USA) for 2 h and washed and replenished with DMEM-Ham's F12 medium. Cytotoxicity for all studies was assayed 48 h later by incubating the cells with 3-(4,5-dimethylthiazol-2-yl)-2,5-diphenyltetrazolium bromide (MTT) as previously described (Kaunitz et al, 1993). The 48 h time point was chosen to mimic the clinical situation, as the first signs of cisplatin nephrotoxicity become apparent after 48 h in vivo through increased serum creatinine levels. Briefly, the cell monolayers were incubated for 3 h with MTT at a final concentration of 1 mg $ml^{-1}$ in the culture medium and then extracted with 100% 2-propanol. MTT incorporation and metabolism to formazan dye is a measure of cell viability. The concentration of formazan dye was determined spectrophotometrically at 570 nm. The corresponding $IC_{50}$ (drug concentration at 50% growth inhibition) was calculated by linear regression of the resulting logarithmic–linear dose–response curves.

### Cellular accumulation of platinum

For each data point, three confluent 100-mm dishes were exposed to various concentrations of cisplatin or carboplatin for various time periods up to 1 h. For high cisplatin concentrations, crystalline cisplatin obtained from Sigma was weighed out and transferred directly into an appropriate volume of medium. Cisplatin dissolved with vigorous shaking at 37°C within 30 min. The uptake rate of both drugs was linear over a 1-h time range, as shown in Figure 5. After incubation, cell monolayers were rinsed once with ice-cold phosphate-buffered saline (PBS), then trypsinized, detached by gentle shaking, transferred to a test tube and centrifuged for 3 min at 2000 ***g*** at 4°C. The pellet was resuspended in 300 μl of PBS and an aliquot of 30 μl was taken for protein determination. After recentrifugation for 3 min at 2000 ***g***, the pellet was digested with 270 μl of 65% nitric acid overnight and assayed for elemental platinum content by atomic absorption spectroscopy (Parti and Wolf, 1990). The molar amount of elemental platinum is equivalent to the molar amount of incorporated cisplatin or carboplatin. The instrument settings for a Perkin Elmer 2380 atomic absorption spectrophotometer with attached HGA-2100 controller were selected as follows: drying temperature, 75°C; drying time, 50 s; charring temperature, 1800°C; charring time, 40 s; atomizing temperature, 2700°C; atomizing time, 6 s; absorption, 265.9 nm; sensitivity, ± 0.3 μM platinum in sample; detection minimum, 0.2 μM platinum in sample.

### Extraction and electrophoresis of DNA

Cells were treated with 50 μM cisplatin for 2 h, then washed and replenished with complete medium. After 48 h, cells were harvested, washed twice with PBS and lysed in 10 mM Tris-HCl (pH 7.5), 100 mM EDTA, 0.5% sodium dodecyl sulphate (SDS) and 100 μg $ml^{-1}$ proteinase K for 18 h at 37°C. DNA was extracted twice with phenol–chloroform–isoamyl alcohol (25:24:1) (PCI) and recovered in the aqueous phase. DNA was precipitated with ethanol, centrifuged for 30 min at 10 000 ***g***, and incubated in TE buffer [10 mM Tris-HCl (pH 8), 1 mM EDTA] containing 100 μg $ml^{-1}$ RNAase for at least 1 h at 37°C. DNA was extracted one more time in PCI and precipitated in 70% ethanol. It was then resuspended in TE buffer and 10-μg DNA samples were electrophoresed on a 1% agarose gel for 2 h at 45 V. Negatives of Polaroid pictures of the gel were scanned with an AMBIS optical imaging system per video camera and, using AMBIS QuantProbe software, the areas of cleaved vs uncleaved DNA were quantified densitometrically.

### DAPI staining of nuclear DNA

The fluorescent dye DAPI (4′,6-diamidine-2′-phenylindole dihydrochloride, Boehringer Mannheim Biochemical) stains nuclear DNA so that condensation and division of chromatin can be readily detected. Cells were grown to subconfluence on coverslips and exposed to 50 μM cisplatin for 2 h. After washing and refeeding with fresh medium, cells were incubated for an additional 48 h. They were then incubated with DAPI–methanol (1 μg $ml^{-1}$) for 15 min at 37°C. Cells were washed once with methanol and then air dried on the coverslip. Apoptotic cells were easily identified and the per cent apoptosis was determined by examination of at least 300 nuclei. Pictures were taken with an inverted microscope–camera apparatus with a 340-nm excitation filter and a 400-nm barrier filter at a magnification of × 400.

### Western blot analysis

Following trypsinization of confluent cell layers, cells were washed in cold PBS and lysed for 10 min on ice in 10 μl of lysis buffer [1% Triton-X 100 (Sigma), 0.5% NP40, 10 mM Tris, pH 7.4, 150 mM sodium chloride, 1 mM EDTA, 1 mM EGTA, 0.4 mM sodium vanadate, 0.2 mM sodium fluoride, 0.4 mM phenylmethylsulphonyl fluoride, 1.4 μg $ml^{-1}$ pepstatin, 25 mM *p*-nitrophenyl phosphate]. Lysates were cleared at 14 000 r.p.m. in a microcentrifuge for 15 min at 4°C. Protein (30 μg) from each sample was boiled for 5 min in loading buffer (50 mM Tris, pH 6.8, 2% SDS, 10% glycerol, 0.1% bromophenol blue, 1% β-mercaptoethanol). Proteins were separated by 12.5% SDS-polyacrylamide gel electrophoresis and transferred onto PVDF membranes (Bio-Rad Trans-Blot Transfer Medium). The membranes were blocked for 1 h at room temperature in 3% bovine serum albumin, 5% non-fat dried milk in TBS buffer (10 mM Tris pH 7.5, 100 mM sodium chloride, 0.2% Tween 20). After four washes in TBS, the membranes were incubated with 0.5 μg $ml^{-1}$ rabbit anti-mouse BCL-2, BCL-X and BAX antibodies (kind gift of Dr John Reed, La Jolla, CA, USA) for 1 h. After six more washes, the membranes were exposed to 1 μg $ml^{-1}$ horseradish peroxidase (HRP)-labelled goat anti-rabbit IgG antibody (Amersham, Arlington Heights, IL, USA) and the bands of the BCL family proteins were detected with an ECL system.

### Statistics

For cytotoxicity studies, the data were based on at least three independent experiments for a five point dose–response curve in triplicate. The $IC_{50}$ values for each cell line were determined by linear regression of the corresponding logarithmic–linear dose–response

© Cancer Research Campaign 1999

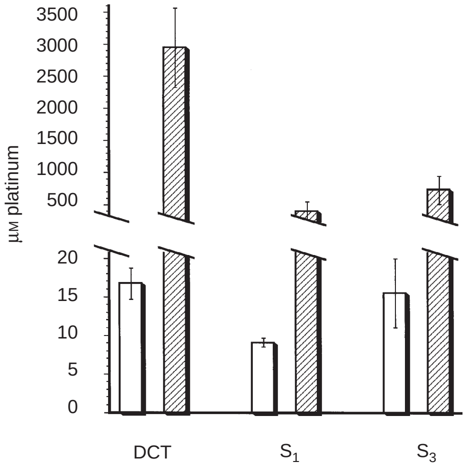

**Figure 1** $IC_{50}$ values for cisplatin (□) and carboplatin (▨) on DCT, $S_1$ and $S_3$ cells ($IC_{50}$, dose level resulting in 50% inhibition of cell survival). Confluent cell monolayers were treated with a range of platinum concentrations for 2 h. Survival was determined by MTT assay after 48 h. The $IC_{50}$ values were determined by linear regression of the corresponding log-linear dose–response curves

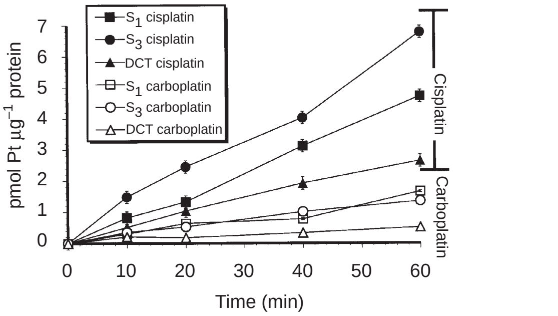

**Figure 2** Time course of cisplatin and carboplatin uptake in DCT, $S_1$ and $S_3$ cells. Confluent cell monolayers were exposed to 100 µM platinum for various periods. The elemental platinum content was measured by flameless atomic absorption spectroscopy and correlated with the protein content of the samples

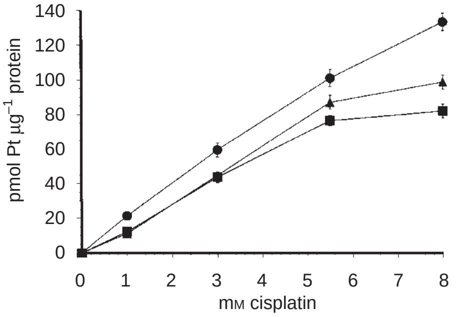

**Figure 3** Uptake of cisplatin as a function of concentration in DCT (▲), $S_1$ (■) and $S_3$ (●) cells. Confluent cell monolayers were exposed to various platinum concentrations up to 8 mM for 1 h. The elemental platinum content was measured by flameless atomic absorption spectroscopy and correlated with the protein content of the samples curves. The means and the population standard deviations of the $IC_{50}$ values were calculated using standard single-variable statistics. Variability is expressed in means and standard deviations. Data of each treatment group were subject to a one-way analysis of variance. Group comparisons were done with a Student–Newman–Keuls test with significance taken at $P < 0.05$.

### RESULTS

### Platinum toxicity in renal tubule epithelial cells

To determine drug cytotoxicity, confluent renal tubule epithelial (RTE) cell layers were exposed to increasing concentrations of cisplatin or carboplatin for 2 h and then washed. Monolayers were refed with fresh medium and 48 h later an MTT assay was performed. Figure 1 shows the differential sensitivity of $S_1$, $S_3$ and DCT cell lines to cisplatin and carboplatin. The $IC_{50}$ values for cisplatin were 9.07 ± 0.6 µM for $S_1$ cells, 15.5 ± 4.5 µM for $S_3$ cells and 16.7 ± 2 µM for DCT cells. The $IC_{50}$ for $S_1$ cells was significantly lower ($P = 0.035$) than that of the $S_3$ and DCT cell lines. Thus, $S_1$ cells were approximately 1.8-fold more sensitive to cisplatin than $S_3$ and DCT cells. All three cell lines were much less sensitive to carboplatin. The corresponding $IC_{50}$ values for carboplatin were 390 ± 150 µM for $S_1$ cells, 730 ± 200 µM for $S_3$ cells and 2940 ± 600 µM for DCT cells. Again, the $S_1$ cells were the most sensitive to carboplatin ($P < 0.05$). However, DCT cells were distinctly more resistant to carboplatin than were $S_3$ cells ($P < 0.05$).

### Platinum drug uptake studies

The comparative uptake studies were performed with a concentration of 100 µM for all compounds. A cisplatin concentration of 100 µM was chosen because it resulted in significant cytotoxicity at 48 h and because it approximated urinary cisplatin concentrations that are associated with nephrotoxicity (Guo, 1992). Urinary platinum may significantly contribute to nephrotoxicity because of a reabsorption of cisplatin into the nephron (see also Discussion). Figure 2 shows the time course of cisplatin and carboplatin uptake in DCT, $S_1$ and $S_3$ cells. Platinum uptake in all three cell lines was linear for up to 1 h. After a 1-h exposure to 100 µM cisplatin, $S_1$, $S_3$ and DCT cells took up cisplatin at different rates: $S_1$ cells accumulated 4.73 ± 0.2, $S_3$ cells 6.8 ± 0.2 and DCT cells 2.65 ± 0.2 pmol $h^{-1}$ $\mu g^{-1}$ protein ($P < 0.05$). Thus, each cell line displayed distinct differences in the rates of cisplatin accumulation. The relative differences in the rates of accumulation did not correlate with relative differences in cisplatin cytotoxicity among the cell lines. For example, $S_1$ cells were found to be most sensitive to cisplatin-induced cytotoxicity whereas $S_3$ cells accumulated cisplatin at the highest rate.

Carboplatin was taken up at a significantly slower rate than cisplatin. After a 1-h exposure the carboplatin levels were 1.66 ± 0.03 in $S_1$ cells, 1.35 ± 0.01 in $S_3$ cells and 0.51 ± 0.03 pmol $h^{-1}$ $\mu g^{-1}$ protein in DCT cells ($P < 0.05$). The rates of carboplatin accumulation were three- to fivefold lower than the corresponding rates of cisplatin accumulation but could not fully account for the 43- to 176-fold lower cytotoxicity of carboplatin compared with cisplatin. The rates of uptake of carboplatin roughly correlated with carboplatin cytotoxicity in the three cell lines such that $S_1$ cells, which proved to be the most sensitive to carboplatin, also accumulated it at the highest rate, whereas DCT cells were the most resistant to carboplatin and accumulated it at the lowest rate.

The uptake rate was studied as a function of cisplatin concentration, and the results are shown in Figure 3. Uptake studies by other investigators cited herein (Binks and Dobrota, 1990; Mann et al,

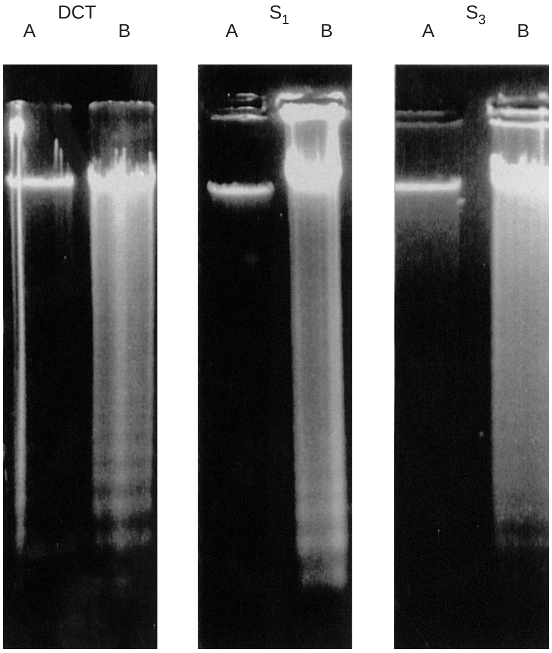

**Figure 4** DNA electrophoresis of control cells (lane A) and 48 h after cisplatin treatment (50 μM cisplatin for 2 h, lane B). From left to right: DCT cells from the distal convoluted tubule, $S_1$ cells from an early portion of the proximal tubule and $S_3$ cells from a late portion of the proximal tubule

1990), were based on the assumption that the solubility limit of cisplatin is 3.33 mM (1 mg $ml^{-1}$), the concentration used by Bristol-Myers Squibb for infusible cisplatin preparations. However, solubility of cisplatin is cited in the Merck Index with 2.53 mg $ml^{-1}$ in water at 25°C, which corresponds to 8.43 mM. A plot of uptake rate as a function of concentrations up to 8 mM revealed a saturable component to cisplatin uptake. $S_1$ and DCT cells showed a non-linear rate vs concentration curve, suggesting that a saturable uptake mechanism may be involved. $S_3$ cells also showed a slightly curved line, however saturation was less clear in this cell line. As it has long been hypothesized that cisplatin uptake occurs through simple diffusion as well as through a gated channel (Binks and Dobrota, 1990), we applied a non-linear regression program to the data. With this curve-fit model, the resulting curve approximating Michaelis–Menten kinetics explained 99% of the experimental data. The three cell types showed distinct differences in their mechanism of uptake in terms of the apparent $V_{max}$ and $K_M$ for transport. The $S_3$ cells, which consistently displayed the highest uptake rate, had an apparent $V_{max}$ of 770 pmol $h^{-1}$ $\mu g^{-1}$ protein and an apparent $K_M$ of 38 mM, suggesting a high-capacity, low-affinity transport mechanism. The DCT cells had an apparent $V_{max}$ of 250 pmol $h^{-1}$ $\mu g^{-1}$ protein and an apparent $K_M$ of 11 mM, and $S_1$ cells had an apparent $V_{max}$ of 180 pmol $h^{-1}$ $\mu g^{-1}$ protein and an apparent $K_M$ of 8 mM. The lower apparent $K_M$ and $V_{max}$ values for DCT and $S_1$ cells in comparison with $S_3$ cells suggest a lower capacity but higher affinity transport process.

### Presence of apoptosis and expression of BCL family proteins

The role of apoptosis in cisplatin-induced cell death was assessed after treating confluent cell monolayers with 50 μM cisplatin for 2 h. DNA fragmentation and cellular–nuclear morphology were assessed 48 h later. Gel electrophoresis of isolated DNA (Figure 4) demonstrated the formation of endonucleosomal DNA fragmentation in all three cell lines 48 h after cisplatin treatment. Densitometric analysis demonstrated 79 ± 7% fragmented DNA in $S_1$ cells, 76 ± 6% fragmentation in $S_3$ cells and 81 ± 9% fragmentation in DCT cells. Figure 5 shows morphological changes in DAPI-stained nuclei of $S_3$ cells after cisplatin treatment. The nuclei of the two other cell lines displayed similar characteristics (not shown). Compared with the control group of non-treated cells (left), the nuclei of the treated group were smaller and the chromatin was segregated into hypercondensed domains, which assumed a sharply defined spherical or crescentic shape in some cells (right, see arrows). For 50 μM cisplatin treatment for 2 h, assayed after 48 h, the count of at least 300 apoptotic vs non-apoptotic nuclei revealed the following proportion of apoptotic nuclei: 42 ± 7% for $S_1$, 28 ± 5% for $S_3$ and 37 ± 9% for DCT cells.

To investigate the expression pattern of BCL family proteins, protein extracts of $S_1$, $S_3$ and DCT cells were assayed by Western blot analysis. All three cell lines expressed very low amounts of BCL-2 protein relative to BAX protein (Figure 6), with $S_3$ cells expressing slightly more than the other cell lines. However, the most marked difference was seen in BCL-X expression, with $S_1$ cells expressing considerably less protein than either $S_3$ or DCT cells (15% vs $S_3$ and DCT cells by densitometry). In contrast, BCL-X expression was considerably lower in $S_1$ cells than in $S_3$ and DCT cells. BCL-X immunoblotting detected a 31-kDa molecular weight band consistent with the long form of BCL-X, the anti-apoptotic protein BCL-$X_L$. Although our anti-BCL-X antibody can also react with the pro-apoptotic short form, BCL-$X_s$, we did not detect the expression of BCL-$X_S$ in our immunoblots.

### DISCUSSION

These results indicate that cytotoxicity and uptake of cisplatin differ in murine renal tubule epithelial cells derived from different segments of the nephron. Our model was designed to mimic the in vivo condition as closely as possible. The available literature suggested that the majority of cisplatin, administered systemically at a high dose of 100 mg $m^{-2}$ over a period of 1 h, is largely cleared via the kidney within a few hours after administration. Toxicological studies also suggest that critical effects to the nephron occur within the first 4 h after administration (Daley-Yates and McBrien, 1984; Mistry et al, 1989). In addition, in vivo renal damage can be detected by increased serum creatinine levels at 48 h. Nephrotoxicity is observed in 70–80% of patients after cisplatin treatment, when urinary platinum levels rise up to 40 μg $ml^{-1}$ (133 μM) or plasma platinum levels are greater than 4 μg $ml^{-1}$ (13 μM) (Guo, 1992). As urinary platinum levels may be of crucial importance in nephrotoxicity because of reabsorption of the drug into the nephron, we took 100 μM cisplatin as a reference point for our studies. By studying confluent renal epithelial cell monolayers, which mimic the nephron epithelial surface, undergoing a 2-h exposure to cisplatin at clinically relevant urinary concentrations, we determined the uptake directly following incubation and the degree of cytotoxicity with an MTT assay after 48 h. We believe these conditions resemble those occurring during acute renal toxicity secondary to cisplatin exposure. In contrast, our model is not necessarily relevant to chronic platinum-induced nephrotoxicity, which may be cumulative over time.

© Cancer Research Campaign 1999

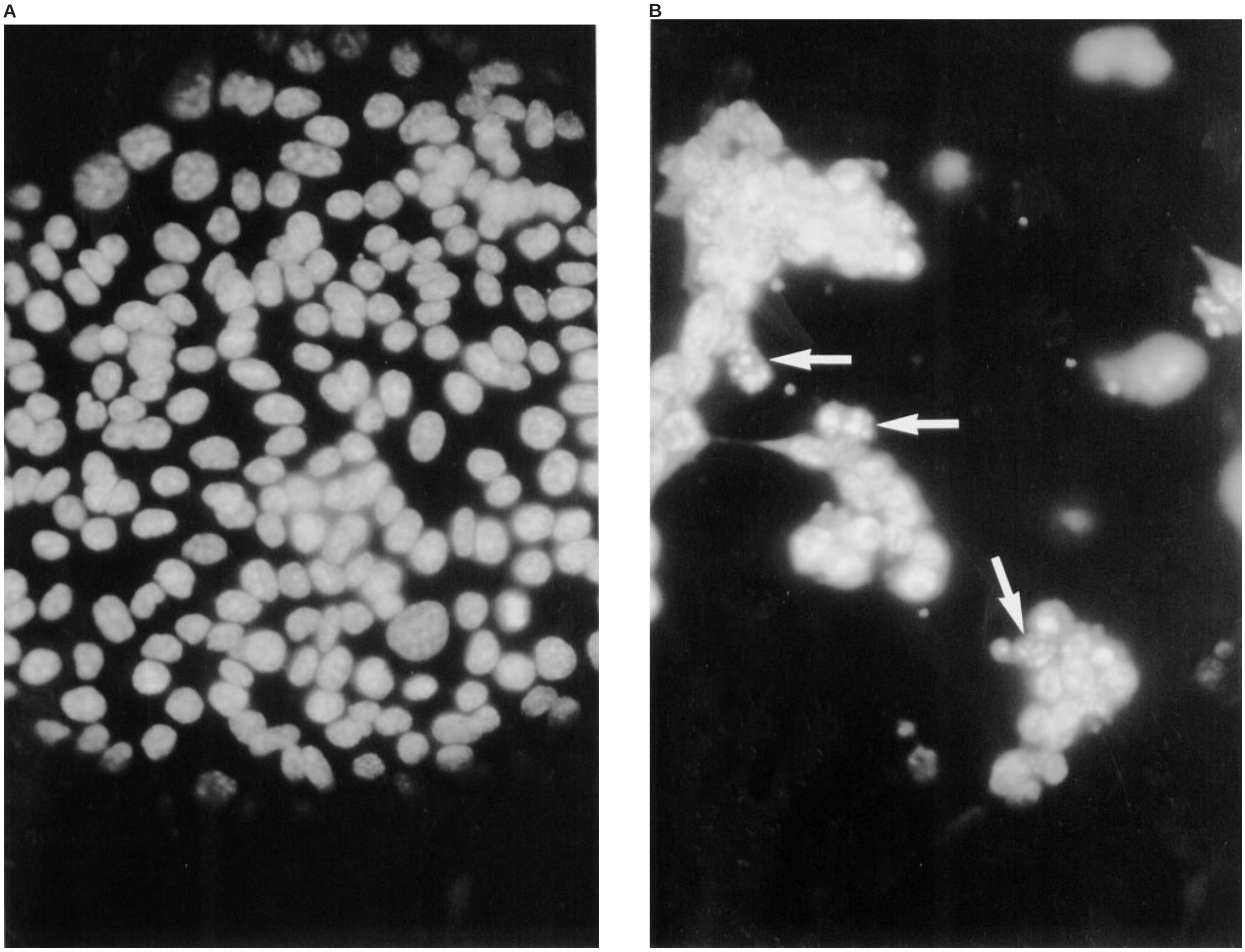

**Figure 5** DAPI staining of $S_3$ control cells (**A**) and 48 h after cisplatin treatment (50 μM cisplatin for 2 h, **B**). Arrows indicate condensed chromatin. $S_1$ and DCT cell lines displayed very similar characteristics. Pictures were taken at a magnification of × 400

Previous in vivo work has demonstrated that proximal tubular segments of the inner cortex and distal convoluted tubular segments of the nephron are injured by cisplatin (Gonzales-Vitale et al, 1977; Blachley and Hill, 1981; Chopra et al, 1982). Our cell line data correlate with the in vivo situation in that $S_1$ cells of the early proximal tubule were most sensitive to the cisplatin cytotoxicity, followed by $S_3$ cells of the late proximal tubule and DCT cells from the distal convoluted tubule.

The differential cytotoxic effects of cisplatin among the three different cell lines were not due to differential uptake rates. Although differences in uptake among the $S_1$, $S_3$ and DCT cell lines were observed, those differences did not correlate with differences in cytotoxicity. Uptake rates were highest in $S_3$ cells, lowest in DCT cells and intermediate in $S_1$ cells. Therefore it appeared that differences in the transport processes among the cell lines could not explain the observed differential sensitivity to cisplatin. However, it is still possible that differential uptake in vivo could play a role in differential cytotoxicity because the distribution of cisplatin accumulation along the nephron roughly correlates with areas of damage (Gonzales-Vitale et al, 1977; Blachley and Hill, 1981; Chopra et al, 1982).

Cisplatin causes apoptosis in cancer (Dive and Hickman, 1991) and kidney cells (Lieberthal et al, 1996). The differential cytotoxicity among the three cell lines may be related to different sensitivities of the cell lines to apoptotic cell death. Our data demonstrate that cell death from cisplatin occurs through apoptosis in $S_1$, $S_3$ and DCT cells. A recent in vitro study demonstrated that the mechanism of renal tubule epithelial cell death was determined by the concentration of cisplatin. A low concentration (8 μM) induced apoptotic death, whereas a high concentration (800 μM) resulted in necrosis (Lieberthal et al, 1996). However, renal cells are never exposed in vivo to such high necrosis-inducing concentrations (Guo, 1992). Our findings clearly demonstrate that renal cell cytotoxicity induced by cisplatin concentrations and exposure times that resemble the clinical situation is due to the induction of apoptosis. Thus, the relative expression of proteins in the BCL family may regulate cisplatin cytoxicity and may account for the lack of correlation between the uptake of cisplatin and cytotoxicity (Miyashita and Reed, 1992; Minn et al, 1995). Although we assessed the expression of only three proteins in this rapidly expanding family, it was interesting that $S_1$ cells, which had an increased sensitivity to the cytotoxic effect of cisplatin, had a rela-

© Cancer Research Campaign 1999

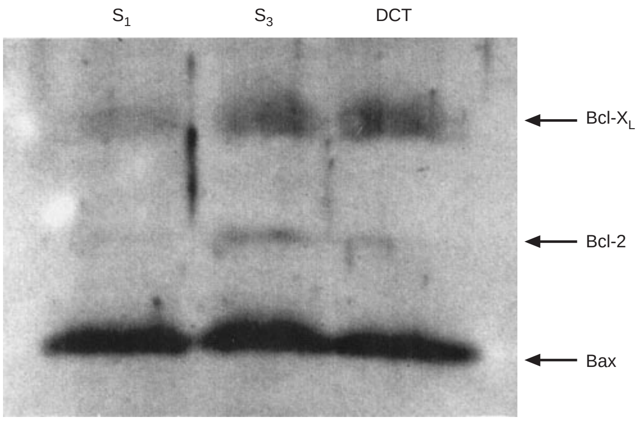

**Figure 6** Western blot analysis of BCL-2, BAX and BCL-X in renal cell lines. Thirty micrograms of protein extracted from $S_1$, $S_3$ and DCT cell lines was electrophoresed and immunostained with anti-BCL-2, -BAX and BCL-X antibodies tively reduced expression in the anti-apoptotic BCL-$X_L$ protein. The reduced expression of the anti-apoptotic BCL-$X_L$ protein may have a greater impact on apoptosis than the reduction of the anti-apoptotic BCL-2 protein (Simonian et al, 1997). This may explain the similar cytotoxic effects of cisplatin in $S_3$ and DCT cells despite reduced expression of BCL-2 in DCT cells compared with $S_3$ cells.

Although differences in uptake rates of cisplatin did not explain the differential cytotoxicities observed among the three cell lines, the differences in uptake characteristics suggest that different transport mechanisms for cisplatin occur in the different cell lines. In vivo studies have demonstrated that proximal tubule cells of the inner cortex and outer medulla and distal convoluted cells accumulate cisplatin (Gonzales-Vitale et al, 1977; Blachley and Hill, 1981; Chopra et al, 1982). Our data in cultured cells correlate with these in vivo data. Furthermore, the cell culture model allowed a better quantitative characterization of the cisplatin uptake process because of the ability to control the concentrations of cisplatin and the timing of exposure, and to measure accurately cisplatin accumulation in the cultured cells. Until recently, the strongest argument against a transporter-mediated cisplatin uptake mechanism was that cisplatin uptake was not saturable. Previous uptake studies were performed at lower cisplatin concentrations because of the assumption that the solubility limit of cisplatin in saline was 3.3 mM (Binks and Dobrota, 1990; Mann et al, 1990). Our experiments demonstrated solubility at 10 mM at 37°C. Studying the uptake of cisplatin over a range of concentrations up to 8 mM allowed us to obtain evidence that a component of cisplatin uptake was saturable in $S_1$ and DCT cells. Because of the high apparent $K_M$ and the limitations of solubility of cisplatin, it was not possible to separate completely the saturable from the non-saturable components of cisplatin uptake. Thus, although the regression analysis demonstrated that Michaelis–Menten-type kinetics could largely explain the experimental data, we prefer to refer to the calculated $K_M$ and $V_{max}$ values as 'apparent' values. Even with these reservations, the data suggested that the cell lines displayed differences in uptake mechanisms. The capacity for cisplatin uptake was higher in $S_3$ cells than in $S_1$ and DCT cells, as was suggested by higher apparent $V_{max}$ values in $S_3$ cells. The similarity in the apparent $K_M$ values observed in $S_1$ and DCT cells suggested that the affinity for cisplatin was similar in these two cell types, whereas the higher $K_M$ value in $S_3$ cells suggested a reduced affinity for cisplatin.

It is well known that cisplatin is much more nephrotoxic in vivo than is carboplatin. In our study, all three renal cell lines were much more sensitive to cisplatin than to carboplatin, with a 43- to 176-fold difference in sensitivity. This large relative difference in toxicity in the renal cells contrasted with data on various tumour cell lines (Knox et al, 1986; Hospers et al, 1988; Schurig et al, 1990; Mellish et al, 1993; Klaushofer et al, 1995) which displayed only a 5- to 24-fold greater sensitivity to cisplatin compared with carboplatin. In experiments not presented here, we also compared the immortalized, non-transformed RTE cell lines with non-renal control, murine immortalized, non-transformed osteoblasts, MC3T3 cells (Micetich et al, 1985). MC3T3 cells showed a 20- to 30-fold difference in sensitivity between cisplatin and carboplatin, i.e. similar to the tumour cells. These data suggest that renal cells exhibit properties that render them more sensitive to cisplatin, or less sensitive to carboplatin, when compared with tumour and other non-renal cells.

The difference in the cytotoxic effects between cisplatin and carboplatin observed in the three renal cell lines could not entirely be accounted for by differences in uptake because cisplatin had only a three- to fivefold higher uptake rate than carboplatin. Thus, mechanisms other than transport appeared to be involved in the differential toxicities of cisplatin and carboplatin. One possible explanation for the different renal cytotoxicities of cisplatin and carboplatin is that in renal cells cisplatin tends to form more toxic, reactive intermediates (e.g. aquated compounds) than carboplatin (Knox et al, 1986; Micetich et al, 1985). Because cisplatin has demonstrated a higher anti-tumour activity than carboplatin in most tumour models, the major use of carboplatin has been when nephrotoxicity is a major risk, such as in high-dose chemotherapy with bone marrow stem cell rescue. Thus, an understanding of the differences between these drugs that influence nephrotoxic potential might help in future studies that attempt to ameliorate cisplatin side-effects.

In summary, the use of homogeneous cell lines derived from different portions of the nephron allowed us to detect significant differences in platinum cytotoxicity and uptake among the cell lines. These data suggest that future therapeutic interventions to protect renal tubular epithelial cells from cisplatin toxicity will have to take into consideration the different sensitivities and transport characteristics displayed by cells from different portions of the nephron.

### ACKNOWLEDGEMENTS

This work was supported by research funds from the Department of Veterans Affairs, the Jonsson Comprehensive Cancer Center and the Cancer Research Coordinating Committee of the University of California. D Katz was a recipient of an American Heart Association, California Affiliate Student Research Award.

### REFERENCES

Binks SP and Dobrota M (1990) Kinetics and mechanics of uptake of platinum-based pharmaceuticals by the rat small intestine. *Biochem Pharmacol* **40**: 1329–1336

© Cancer Research Campaign 1999

Blachley JD and Hill JB (1981) Renal and electrolyte disturbances associated with cisplatin. *Ann Int Med* **95**: 628–632

Brady HR, Kone BC, Stromski ME, Zeidel ML, Giebisch G and Gullans SR (1990) Mitochondrial injury: an early event in cisplatin toxicity to renal proximal tubules. *Am J Physiol* **258**: F1181–F1187

Chopra S, Kaufman JS, Jones TW, Hong WK, Gehr MK, Hamburger RJ and Trump BF (1982) Cis-diamminedichloroplatinum-induced acute renal failure in the rat. *Kidney Int* **21**: 54–64

Colvin M (1993) Alkylating agents and platinum antitumor compounds. In *Cancer Medicine*, Holland JF, Frei E III, Bast RC Jr, Kufe DW, Morton DL and Weichselbaum RR (eds), pp. 733–754. Lea & Febiger: Philadelphia

Daley-Yates PT and McBrien DC (1984) Cisplatin metabolites in plasma, a study of their pharmacokinetics and importance in the nephrotoxic and antitumour activity of cisplatin. *Biochem Pharmacol* **33**: 3063–3070

Dive C and Hickman JA (1991) Drug–target interactions: only the first step in the commitment to a programmed cell death? *Br J Cancer* **64**: 192–196

Fady C, Gardner A, Jacoby F, Briskin K, Tu Y, Schmid I and Lichtenstein AK (1995) Atypical apoptotic cell death induced in L929 targets by exposure to tumor necrosis factor. *J Interferon Cytokine Res* **15**: 71–80

Gonzalez-Vitale JC, Hayes DM, Cvitkovic E and Sternberg SS (1977) The renal pathology in clinical trials of cis-platinum (II) diamminedichloride. *Cancer* **39**: 1362–1371

Guo JH (1992) Relationship between plasma and urinary platinum pharmacokinetics with cisplatin nephrotoxicity in breast cancer patients. *Chung-Hua Chung Liu Tsa Chih Chinese J Oncol* **14**: 150–153

Hospers GAP, Mulder NH, de Jong B, de Ley L, Uges DRA, Fichtinger-Schepman AMJ, Scheper RJ and de Vries EGE (1988) Characterization of a human small cell lung carcinoma cell line with acquired resistance to cis-diamminedichloroplatinum(II) *in vitro*. *Cancer Res* **48**: 6803–6807

Kaunitz JD, Cummins VP, Mishler D and Nagami GT (1993) Inhibition of gentamicin uptake into cultured mouse proximal tubule epithelial cells by L-lysine. *J Clin Pharmacol* **33**: 63–69

Klaushofer K, Varga F, Glantschnig H, Fratzl-Zelman N, Czerwenka E, Leis HJ, Koller K and Peterlik M (1995) The regulatory role of thyroid hormones in bone cell growth and differentiation. *J Nutrition* **125** (suppl. 7): 1996S–2003S

Knox RJ, Freidlos F, Lydall DA and Roberts JJ (1986) Mechanism of cytotoxicity of anticancer platinum drugs: evidence that cis-diamminedichloroplatinum (II) and cis-diammine (1,1-cyclobutanedicarboxylate) platinum (II) differ only in the kinetics of their interaction with DNA. *Cancer Res* **46**: 1972–1979

Leibbrandt MEI, Wolfgang GHI, Metz AL, Ozobia AA and Haskins JR (1995) Critical subcellular targets of cisplatin and related platinum analogs in rat renal proximal tubule cells. *Kidney Int* **48**: 761–770

Lieberthal W, Triaca V and Levine J (1996) Mechanisms of death induced by cisplatin in proximal tubular epithelial cells: apoptosis vs. necrosis. *Am J Physiol* **270**: F700–F708

Loehrer PJ and Einhorn LH (1984) Cisplatin. *Ann Int Med* **100**: 704–713

Mann SC, Andrews PA and Howell SB (1990) Short-term cis-diamminedichloroplatinum (II) accumulation in sensitive and resistant human ovarian carcinoma cells. *Cancer Chemother Pharmacol* **35**: 236–240

Mellish KJ, Kelland LR and Harrap KR (1993) In Vitro platinum drug chemosensitivity of human cervical squamous cell carcinoma cell lines with intrinsic and acquired resistance to cisplatin. *Br J Cancer* **68**: 240–250

Micetich KC, Barnes D and Erickson LL (1985) A comparative study of the cytotoxicity and DNA-damaging effects of cis-(diammino) (1,1-cyclobutanedicarboxylate)-platinum (II) and cis-diamminedichloroplatinum (II) on L1210 cells. *Cancer Res* **45**: 4043–4047

Minn AJ, Rudin CM, Boise L Thompson CB (1995) Expression of BCL-X-L can confer a multidrug resistance phenotype. *Blood* **86**: 1903–1910

Mistry P, Lee C and McBrien DC (1989) Intracellular metabolites of cisplatin in the rat kidney. *Cancer Chemother Pharmacol* **24**: 73–79

Miyashita T and Reed JC (1992) BCL-2 gene transfer increases relative resistance of S49.1 and WEHI 7.2 lymphoid cells to cell death and DNA fragmentation induced by glucocorticoids and chemotherapeutic drugs. *Cancer Res* **52**: 5407–5411

Nagami GT, Warech EM and Mischler DR (1990) Cells derived from S1, S2 and S3 proximal tubule segments of a transgenic mouse. *J Am Soc Nephrol* **1**: 656

Nagami GT, Warech EM and Mischler DR (1991) Effect of angiotensin II on ammonia production by mouse S1 and S3 proximal tubule cell lines. *J Am Soc Nephrol* **2**: 707

Nagami GT, Warech EM and Mischler DR (1992) Ammonia production and transport by cultured proximal tubule cells grown on permeable supports. *J Am Soc Nephrol* **3**: 783

Palmiter RD, Chen HY, Messing A and Brinster RL (1985) SV 40 enhancer and large-T antigen are instrumental in development of choroid plexus tumors in transgenic mice. *Nature* **316**: 457–460

Parti R and Wolf W (1990) Quantitative subcellular distribution of platinum in rat tissues following i.v. bolus and i.v. infusion of cisplatin. *Cancer Chemother Pharmacol* **26**: 188–192

Schurig JE, Rose WC, Catino JJ, Gaver RC, Long BH, Madissoo H and Canetta R (1990) The Pharmacologic Characteristics of Carboplatin: Preclinical Experience. In *Carboplatin (JM-8), Current Perspectives and Future Directions*, Bunn PA, Canetta R, Ozols RF and Rosencweig M (eds), pp. 3–17. WB Saunders: Philadelphia

Simonian PC, Grillot DAM and Nunez G (1997) BCL-2 and BCL-XL can differentially block chemotherapy induced cell death. *Blood* **90**: 1208–1216

© Cancer Research Campaign 1999
